# An ancient genome duplication contributed to the abundance of metabolic genes in the moss *Physcomitrella patens*

**DOI:** 10.1186/1471-2148-7-130

**Published:** 2007-08-02

**Authors:** Stefan A Rensing, Julia Ick, Jeffrey A Fawcett, Daniel Lang, Andreas Zimmer, Yves Van de Peer, Ralf Reski

**Affiliations:** 1 Plant Biotechnology, Faculty of Biology, University of Freiburg, Schaenzlestr. 1, D-79104 Freiburg, Germany.; 2 Department of Plant Systems Biology, VIB, B-9052 Ghent, Belgium.; 3 Bioinformatics and Evolutionary Genomics, Department of Molecular Genetics, Ghent University, Technologiepark 927, B-9052 Ghent, Belgium.

## Abstract

**Background:**

Analyses of complete genomes and large collections of gene transcripts have shown that most, if not all seed plants have undergone one or more genome duplications in their evolutionary past.

**Results:**

In this study, based on a large collection of EST sequences, we provide evidence that the haploid moss *Physcomitrella patens *is a paleopolyploid as well. Based on the construction of linearized phylogenetic trees we infer the genome duplication to have occurred between 30 and 60 million years ago. Gene Ontology and pathway association of the duplicated genes in *P. patens *reveal different biases of gene retention compared with seed plants.

**Conclusion:**

Metabolic genes seem to have been retained in excess following the genome duplication in *P. patens*. This might, at least partly, explain the versatility of metabolism, as described for *P. patens *and other mosses, in comparison to other land plants.

## Background

In contrast to animals, the entire multicellular diploid generation of plants (along with the cuticle and thick-walled, non-motile spores) probably evolved after the transition to land [1,2]. All land plants display alternating multicellular generations – the sexual, haploid gametophyte and the asexual, diploid sporophyte. In early land plant fossils the gametophytic and sporophytic generation share about equal morphological complexity, making it likely that the gametophyte was reduced and the sporophyte became the dominant generation in vascular plants [1-3] while in “bryophytes” (mosses, hornworts and liverworts) the sporophyte generation was reduced and the gametophyte became dominant. Thus, “bryophytes” in comparison with vascular plants enable inference of early states of land plant evolution. Based upon spores found in the fossil record, the first plants had occupied the land in the Middle Ordovician, approximately 460 million years ago (MYA) [1]. The first splits among the *Embryophyta *separated the *Bryopsida *(mosses), *Antocerotophyta *(hornworts) and *Marchantiophyta *(liverworts) from the remainder of the land plants, the vascular plants. The oldest liverwort fossils are from the Late Devonian, ~360 MYA, the oldest mosses to be found in the fossil record are from the Permian, ~270 MYA [4,5]. The first deposits containing remnants of modern mosses are from the Jurassic and Cretaceous; based on these fossils some extant species exhibited only limited morphological change in the past 80 MY [5,6]. Most of the mosses deposited in European Miocene (24 MYA) are morphologically identical to extant European genera and even species [5,7]. Mosses embedded in Caribbean amber (20–45 MYA) could also be traced to a large extent to extant genera and species [8]. In summary, some moss species might be 40–80 MY old, whereas some genera might even be 80–100 MY old [6], which is also seconded by recent phylogenetic analyses [9,10].

Gene and genome duplications are a driving force of eukaryotic evolution [11,12]. Angiosperms (flowering plants) are paleopolyploids, i.e. the genome of their common ancestor was subject to a large-scale or even genome-wide duplication event during the Late Jurassic or Early Cretaceous, 100–160 MYA [13,14]. This duplication event might have triggered the angiosperm radiation during the Late Cretaceous, which is apparent in the fossil record [15]. There is evidence for several more large-scale or genome-wide duplication events among the angiosperms. The core eudicots apparently duplicated their genome in the Late Cretaceous, while the common ancestor of the *Brassicales *did so again in the Cenozoic [13,16]. Also poplar, of which the genome sequence has been determined recently, has undergone an additional genome duplication event ~60 MYA, independent of the one in the *Brassicales *[17]. Recently, paleopolyploidy has been suggested for several basal angiosperm species as well as for some gymnosperms [18]. Interestingly, the retention of genes after such large-scale duplication events has been shown to be biased towards certain functional classes [16,19,20] and it has been argued that such biased retention of duplicated genes has been a driving force for morphological complexity, increase in biological diversity and eukaryote adaptive radiation [13,21].

The aims of the current study were: (i) to reveal molecular evidence for genome duplications in non-seed plants, in particular in the moss *Physcomitrella patens*, (ii) to date the duplication event(s), and (iii) to study the possible evolutionary consequences by analyzing the retention of different functional classes of genes.

## Results and discussion

### *Physcomitrella patens *is a paleopolyploid

**Figure 1:**
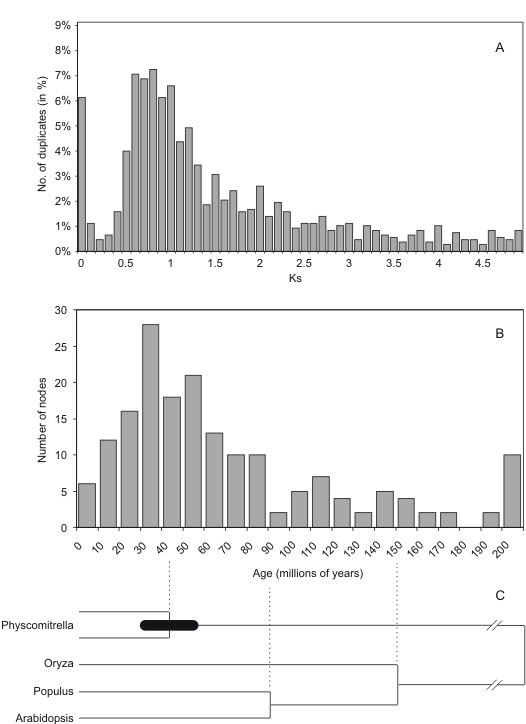
**Dating the genome duplication event in *Physcomitrella patens***. a) Age distribution of paralogous EST sequences. The height of the bars reflects the amount of gene pairs in the respective bin relative to the total amount of K_S _values in the distribution. b) Age distribution of duplicated genes as inferred from their point of divergence relative to the split *Arabidopsis*-poplar or *Arabidopsis*/poplar-rice in a phylogenetic tree as shown in c). The split between *Arabidopsis *and poplar is assumed to have occurred at about 100 MYA, while the split between monocots and eudicots is assumed to have occurred at about 150 MYA.

Based upon a dataset of 24,845 *P. patens *unigenes [22-24], prediction of open reading frames using species-specific models yielded a dataset of 22,237 coding sequences. From those, a total of 2,907 paralogs were determined by all-against-all BLAST searches using previously described parameters [25]. After average linkage clustering, the K_S_-values representing ancient duplication events were calculated. In total, 1,971 genes were placed in 854 clusters. A cutoff distance of K_S _5.0 was used and the K_S_-range was divided into bins of size 0.1. The K_S _age distribution plot exhibits a clearly distinguishable peak at K_S _~0.85, providing evidence for an ancient large-scale or even genome-wide duplication event (Figure 1A). While K_S _values >1.0 should be used with caution because multiple mutations may cause inaccuracies in the estimation [26,27], the K_S _estimate for the peak in the *P. patens *distribution thus probably is trustworthy. Also, while e.g. in *Drosophila *K_S _is lower in genes with strong codon bias, this is not the case for *P. patens *[28] and thus needs not to be dealed with.

In order to obtain additional evidence for a large-scale or genome-wide duplication in *P. patens*, as well as to date this duplication event, we constructed linearized trees (see Methods). We constructed neighbor-joining trees for 487 gene families which contained two to ten *P. patens *genes, one *Chlamydomonas reinhardtii *or *Ostreococcus tauri *gene as an outgroup sequence, and genes from at least two different seed plants (*Arabidopsis thaliana*, poplar, or rice) as reference points. Sequences that were evolving too fast or too slow were removed, after which linearized trees, in which branch lengths are directly proportional to time, were constructed for each gene family [29,30]. This left 330 trees, and after removing nodes with bootstrap values < 70%, we obtained 179 nodes representing the duplication events of *P. patens *in 159 trees that could be used for dating the gene duplications in *P. patens *by comparing the time of duplication with the time of speciation between *A. thaliana *and poplar, assumed 100 MYA [31] and *A. thaliana *or poplar and rice, assumed 150 MYA [32] (see Figure 1B).

Next, we plotted the estimated dates of the 179 *P. patens *duplication events, which are shown in Figure 1C. As can be clearly observed, a majority of the gene duplicates seem to have been created between 30–60 MYA (average 45 MYA), indicating that a large-scale gene duplication or a whole-genome duplication is indeed likely to have occurred around this time. Although using two different calibration dates (100 MYA for the *Arabidopsis*-poplar split, and 150 MYA for the monocot-eudicot split) may affect the age distribution if one of the two calibration dates is unrealistic compared to the other, age distributions obtained for each calibration point separately were very similar (data not shown), suggesting that the dates of 100 MYA and 150 MYA [31,32] are in good agreement with inferred dates from tree topologies.

It should be noted, however, that a number of alternative and more sophisticated methods exist to estimate divergence or duplication dates based on tree inference, even if rate heterogeneity between lineages is present. Yet, as has been shown in several studies (e.g. [33] and references therein), caution should be taken when using such rate-smoothing methods. It is also much harder to process a large amount of data in a high-throughput manner using rate-smoothing methods because of the different parameters that ideally have to be estimated or used for different genes or proteins, while this is not the case when a correction for unequal rates is not required and faster/slower evolving genes are simply removed, as in the linearized trees method applied here. The major disadvantage of the linearized tree method is that a substantial amount of data is omitted from the analysis because trees showing unequal branch lengths for the species under investigation are not considered. However, in the current study there are plenty of data left to provide convincing evidence that a large-scale gene duplication event has occurred in the evolutionary past of *P. patens*. This was the main aim of our study, rather than to come up with the most accurate dating of that event, which will be very difficult anyway given the fact that the calibration points are debateable in the first place.

However, the approximate range of the duplication event is probably trustworthy. If we assume that the genome duplication indeed took place about 45 MYA (average of the peak in Figure 1), and we assume that genes duplicated at that time have an average K_S _value of 0.85 (see Figure 1), we can infer the rate of synonymous substitutions by simply dividing 0.85 by 45 MY. This gives us a rate of 1.9 synonymous substitutions per synonymous site per year, which is very close to the value presented by Koch et al. [34] based on the calibration of molecular clocks for eudicots. However, these substitution rates have to be interpreted with caution, since there are many theoretical and empirical concerns about the accuracy of molecular clocks and the rate of substitutions in different lineages. Some of the major issues are rate heterogeneity in and between lineages caused by evolutionary factors (e.g. generation time), difficulties in interpreting the fossil data used to calibrate the clock, and rate variation among genes, even at synonymous sites.

### An enigma unveiled: ploidy levels and chromosome counts among mosses

Besides the long-term effect of increasing the genetic complexity, there might be several possible short-term advantages of polyploidization events for diploid seed plants (reviewed by [13]), such as heterosis effect, sympatric and allopatric speciation, decreased inbreeding depression and genetic load (allowing selfing populations that can be monoecious and better dispersers). Genome duplication, due to its effects on gene regulation and developmental processes, might also be a foundation for speciation and adaptation through genetic divergence in plants [35]. In the case of the haploid “bryophytes”, however, other effects appear more relevant. The allopolyploidization of dioecious gametophytes might yield a monoecious plant (thus rendering the dispersal of breeding populations easier). A second advantage might be that the duplication of the genome would free the formerly haploid plant from the necessity to preserve the function of crucial single copy genes under all circumstances, thus enhancing the potential for development of new functions. The moss *P. patens *belongs to the *Funariaceae*, is haploid, monoecious and self-fertile. Polyploidization occurs rather frequently during transfection of *P. patens *protoplasts [36]. Among transformants, diploid plants cannot be distinguished from haploid plants using morphological traits alone [37]. The *P. patens *wild type, however, is clearly functionally haploid, as can be seen from segregation ratios [38]. Taken together, our data suggest that the ancestor of *P. patens *underwent polyploidization during the Eocene, potentially becoming hermaphroditic through this process. However, subsequently the plant became functionally haploid again (haploidization) while keeping the duplicated chromosomes. Analogous states are known from seed plants as well, where duplicated chromosomes often remain after allopolyploidization and subsequent diploidization [39,40].

Most liverworts have 9 chromosomes and hornworts usually have 8, 9 or 10; there are few polyploids in both groups. The mosses, in contrast, display chromosome numbers between 4 and 72 [41]. These are probably due to both different base numbers in the different orders and the existence of many aneuploids and polyploids [5]. Within the *Funariaceae*, chromosome counts between 4 and 72 have been reported [41]. While *Funaria hygrometrica *seems to be representative for the majority of *Funariaceae*, *Physcomitrium pyriforme *usually exhibits a higher chromosome count with several samples each being described to contain 18, 26, 36, 52 and 54 haploid chromosomes, with the highest number being 72 chromosomes [41]. Among the analysed *F. hygrometrica *accessions, 51% contain 28 chromosomes. While single accessions were described to contain 4, 21 and 42 chromosomes, the remainder contains either 14 (34%) or 56 (9%) chromosomes. In the case of *P. patens*, chromosome counts of 14 and 28 have been reported for two different isolates [42]. These data make frequent, recent and independent polyploidization events among individual species or genera evident. The haploid chromosome count of the *P. patens *ecotype analysed here is 27 [43], which, given the data presented in this work, would make it a putative paleopolyploid and paleoaneuploid. In a recent phylogenetic analysis, the age of the *Funariales *was determined at ~172 MYA [9]. Therefore, the whole genome duplication analysed here most probably represents a duplication event that occurred after speciation among the *Funariaceae*. Consequently, the different chromosome counts found in extant species like *P. patens*, *F. hygrometrica *and *P. pyriforme *have most probably occurred and been fixed several times independently during evolution. This is further supported by the fact that moss genera or species seem to have become hermaphroditic several times independently during evolution, given the dissipated pattern of mon- and dioecious species within the taxonomic groups [6].

### Gene retention following the whole genome duplication in *P. patens*

It has been demonstrated that retention of functional gene classes after large-scale duplication events is biased in angiosperms. For example, genes involved in signal transduction and transcriptional regulation were preferentially retained after the three whole genome duplication events within the ancestor of *A. thaliana*, while there was selection against retention of these genes after small-scale duplication events [16,19,20]. In order to analyse potential bias among the genes that were retained following the genome duplication in *Physcomitrella patens*, we compared the fractions of genes associated with Gene Ontology (GO) terms [44]. All paralogs with a K_S _of 0.6–1.1 (765 genes) were mapped to GO Slim [45] and compared to the associations of an equally sized random sample. The biological process categories "biosynthesis" and "generation of precursor metabolites and energy" are significantly over-represented (q < 0.05) among the retained paralogs (Table 1). In total, 199 genes, (26%) belong to these categories. Genes of biological process categories that are under-represented within the genome duplication peak are "protein biosynthesis", "organelle organization and biogenesis", "cytoskeleton organization and biogenesis" and "cytoplasm organization and biogenesis". Genes involved in signal transduction and transcriptional regulation, which were preferentially retained after genome duplications in angiosperms [19,20,46], seem not to be retained in excess following the duplication event in *P. patens*. Also, the PFAM domains that were reported to be enriched in plant (*A. thaliana *and rice) duplicate genes [47] were compared with those assigned to genes present in the *P. patens *K_S _peak and were found not to be enriched.

**Table 1:** Over- and under-represented GO categories among genes retained after the *P. patens *genome duplication

**GO category/KO pathway**	**description**	**No. of annotated genes in the peak**	**No. of annotated genes in the reference set**	**fdr corrected p-value**
**enriched**				
GO:0006091	generation of precursor metabolites and energy	80	35	0.0029
GO:0009058	biosynthesis	119	67	0.0109
**reduced**				
GO:0006412	protein biosynthesis	54	82	0.0424
GO:0006996	organelle organization and biogenesis	28	67	0.0005
GO:0007010	cytoskeleton organization and biogenesis	3	19	0.0059
GO:0007028	cytoplasm organization and biogenesis	27	62	0.0010

The genes present in the peak (Figure 1A) representing the whole genome duplication were also mapped against the Kyoto Encyclopedia of Genes and Genomes (KEGG) database in order to analyse in which pathways they are involved. Among the genes that belong to the under-represented GO categories, the lack of ribosomal proteins (48% of those present in the reference set) is noteworthy. The genes belonging to the enriched GO categories all belong to the KEGG ontology (KO) class "metabolism". Among GO:0006091 "generation of precursor metabolites and energy", the major KO pathways are energy and carbohydrate metabolism, among GO:0009058 "biosynthesis", the major KO pathways are carbohydrate, amino acid and lipid metabolism. Thus, both GO and KEGG analyses demonstrate an abundance of genes involved in metabolism that were specifically retained following the whole genome duplication. This is in concordance with the high abundance of metabolic genes in *P. patens *as compared to seed plants which has been described previously [22]. Based on GO mappings of large-scale genome or transcriptome datasets, metabolism-associated transcripts account for 10–44% of seed plant transcriptomes, while their abundance is significantly higher (70–80%) in mosses [22]. Apparently, metabolic genes have been maintained in excess following the large-scale duplication event, which might explain their previously observed abundance in the *P. patens *genome.

### Peculiarities of moss ecology and metabolism

There are species of mosses that can survive long times of dryness (up to 14 years), extreme cold (Antarctica) and heat (70 to 110 degrees Celsius) and are able to prosper in only 0.1% of sun light (while seed plants need at least 2%). In general, mosses are adapted to capture of low light intensities, having low light compensation and saturation points, making use of their one-cell-thick leaflets and lack of waxy cuticles [48]. The structural simplicity of the tissues permits mosses to react immediately to water, CO_2 _and light availability in terms of photosynthesis [48]. Mosses in general are able to grow over a wider temperature range than seed plants, particularly at low temperatures. Many mosses are able to have photosynthetic gain at temperatures as low as -10°C [48]. They typically become dormant in summer heat and drought but are able to immediately photosynthesize upon rehydration. Mosses are able to receive nutrients from the substrate as well as from precipitation and dust [48]. In a multitude of studies, alternative and/or redundant metabolic pathways have been described in mosses. As an example, the reduction of adenosine 5'-phosphosulfate (APS) to sulfite by adenosine 5'-phosphosulfate reductase is considered the key step of sulfate assimilation in seed plants. While APS-reductase is present in *P. patens *as well, this moss also harbors a phosphoadenosine 5'-phosphosulfate (PAPS) reductase, which was previously known e.g. from enteric bacteria [49]. Thus, *P. patens *is able to employ an alternative pathway of sulfate reduction that is not accessible to seed plants. Interestingly, sulfate adenylyltransferase, the enzyme that catalyzes the formation of PAPS from ATP and inorganic sulfate, is encoded by a gene present in the K_S _peak. In *Ceratodon purpureus*. a bifunctional delta-fatty acyl acetylenase/desaturase has been characterized which displays a redundant functionality, being able to introduce a Delta6cis-double bond into 9,12,(15)-C18-polyenoic acids as well as converting a Delta6cis-double bond to a Delta6-triple bond [50]. *P. patens *contains a homolog of the yeast ELO-genes unknown from seed plants, encoding a component of the Delta6-elongase, which is involved in the biosynthesis of C20 polyunsaturated fatty acids [51]. Among the proteins encoded by the K_S _peak genes, 12 are involved in fatty acid metabolism (e.g., fatty acid desaturase, long-chain fatty-acid-CoA ligase), of which eight are involved in fatty acid biosynthesis.

#### Lipid metabolism and volatiles

The tetracyclic diterpene 16alpha-hydroxykaurane is a major lipid compound in *P. patens*, which has been shown to be released into the air [52]. *P. patens *contains high levels of arachidonic acid and lesser amounts of eicosapentaenoic acid, which is due to delta5- and delta6-desaturases that are associated with the synthesis of these fatty acids [53,54]. A complex mixture of fatty acid-derived aldehydes, ketones, and alcohols is released upon wounding of *P. patens*. In contrast to other lipoxygenases cloned so far, the *P. patens *enzyme exhibits an unusually high hydroperoxidase and fatty acid chain-cleaving lyase activity, leading to the formation of unusual oxylipins based on arachidonic acid as substrate [55]. Thus, a highly diverse product spectrum is formed by a single enzyme accounting for most of the observed oxylipins produced by *P. patens*. Also, a *P. patens *gene was cloned and classified to encode an unspecific hydroperoxide lyase having a substrate preference for 9-hydroperoxides of C18-fatty acids [56]. The knockout lines failed to emit (2E)-nonenal while formation of C8-volatiles was not affected, indicating that in contrast to flowering plants the *P. patens *enzyme is involved in formation of a specific subset of volatiles. Ent-kaurene is a precursor for gibberellins (GAs) in plants and fungi. The fungal CPS/KS enzyme catalyzes a two-step reaction corresponding to ent-copalyl diphosphate synthase (CPS) and ent-kaurene synthase (KS) activity in plants. Overexpression of fungal CPS/KS in *A. thaliana *has been shown to rescue GA-deficient phenotypes [57]. Interestingly, the over-expressing plants emitted ent-kaurene as a volatile, inducing airborne action on nearby plants. Recently, an ent-kaurene synthase from *P. patens *was cloned and characterized. The enzyme is a bifunctional cyclase which, like fungal CPS/KS, directly synthesizes ent-kaurene from geranylgeranyl diphosphate [58].

#### Tolerance to abiotic stresses

In comparison with seed plants, *Physcomitrella patens *exhibits a much greater tolerance to abiotic stresses, being able to survive e.g. NaCl concentrations up to 350 mM and sorbitol up to 500 mM [59]. *P. patens *is dehydration tolerant, plants that had lost 92% water on a fresh-weight basis were able to recover successfully [59]. Other mosses, like *Tortula ruralis*, are even desiccation tolerant, the rehydrating gametophytes displaying an abundance of transcripts that code for e.g. enzymes involved in oxidative stress metabolism [60]. The transcript levels of novel putative membrane transporters similar to mammalian inward rectifier potassium channels were shown to be upregulated in *P. patens *upon cold and osmotic stress [61]. The widespread calcifuge moss *Pleurozium schreberi *is moderately tolerant to dissolved SO_2 _(bisulfite). The tolerance mechanism involves extracellular oxidation using metabolic (photo-oxidative) energy, passive oxidation by adsorbed Fe^3+ ^and probably also internal metabolic detoxification [62]. In a comparative classification of alpine mosses, lichens and seed plants, strong illumination caused photodamage in dried leaves, but not in dry moss (*Grimmia alpestris*) and dry lichens [63]. In hydrated mosses, but not in leaves of seed plants, protein protonation and zeaxanthin availability are fully sufficient for effective energy dissipation even when photosystem II reaction centers are open [64]. During desiccation, quenchers accumulate in the poikilohydric moss *Rhytidiadelphus squarrosus *which are stable in the absence of water but revert to non-quenching molecular species on hydration [65]. Together with zeaxanthin-dependent energy dissipation, desiccation-induced thermal energy dissipation protects desiccated poikilohydric mosses against photo-oxidation, ensuring survival during drought periods [65]. There are several proteins encoded by K_S _peak genes that might be related to these phenomena, such as those involved in carotenoid synthesis (1-deoxy-D-xylulose-5-phosphate synthase, zeta-carotene desaturase, two phytoene synthases) and electron transfer (ten light-harvesting complex II chlorophyll a/b binding proteins, two plastocyanins and two cytochrome b6-f complex iron-sulfur subunits).

## Conclusion

It is remarkable that many alternative metabolic pathways exist in *P. patens *and other mosses while they are absent from seed plants. Some of the metabolites produced by these genes, such as volatile lipid compounds, might aid pathogen defence. As an opportunist growing on different types of soil, being able to prosper using a plethora of energy sources might suit *P. patens *well. Drought tolerance is a primordial trait which is especially important for mosses, because they generally do not possess an epidermis or a sturdy cuticula. The biased retention of genes involved in transcriptional regulation and signal transduction in angiosperms, resulting in highly adapted and complex regulatory systems, is likely closely interwoven with their increase in complexity and adaptive radiation [13]. Mosses, however, might follow an entirely different strategy, being generalists rather then specialists in terms of their metabolic gene complement, growing in habitats not readily accessible for seed plants.

## Methods

### Unigene set and ORF prediction

The dataset used consisted of 24,845 unigenes based on ~130,000 public *P. patens *expressed sequence tags (EST), the production parameters of which have been described before [22,23]. An evaluation of several tools for open reading frame (ORF) prediction was carried out, including ESTScan [66], FrameD [67] and Estwise [68]. As it turned out, using *A. thaliana *or plant-trained models yielded a high rate of false positive predictions for *P. patens *genes [23]. Homology-based ORF prediction was hindered by the fact that the closest homologs often shared only 30–40% identity on amino acid level. By dividing all publicly available *P. patens *mRNAs into a training set (226 sequences) and a test set (100 sequences), FrameD turned out to be the most accurate individual tool. After testing, a *P. patens *specific hidden Markov model (HMM) for ESTScan and interpolated HMMs for FrameD were built by using all 326 sequences. In order to improve the quality of the predicted ORFs, FrameD was given the results of a BLASTX-search of the unigenes against Genpept using an E-value cutoff of 1E-10. ORFs were determined by combining the prediction results from ESTScan and FrameD, preferring the latter. In total, 19,313 ORF were detected by FrameD and 21,344 by ESTScan, the combination yielding 22,237 ORF which were used for further analyses.

### Calculating the K_S _distribution

Two Perl scripts were written to identify clusters of paralogous genes and subsequently calculate K_S _distributions. The software ("KeyS") is available upon request. The method used to calculate Figure 1A is described below.

#### Identification of pairs of paralogous genes

To identify similar sequences on peptide level, an all-against-all BLAST-search was performed using BLASTP with an E-value cutoff of 1E-10. Two sequences were defined as paralogs if the sequences could be aligned over a length of at least 150 amino acids and showed at least 30% identity [25]. Gene pairs with a BLAST identity of 98% or higher were further tested for identity because near identical sequences occasionally are present in clustered EST data due to sequencing errors and the fragmentary nature of EST. To do this, the nucleic acid sequences were aligned globally using the EMBOSS [69] implementation of the Needleman-Wunsch algorithm, *needle*. Afterwards all leading and trailing gaps were removed from the alignment. Two sequences were then defined as identical if the aligned sequences had an identity of at least 98.0%. From all identical gene pairs the longer sequence was kept and all gene pairs containing the shorter sequence were discarded.

#### Clustering of paralogous genes

In order to reduce the computational complexity, genes were clustered prior to K_S _calculation. From the list of gene pairs, the genes of the pair with the highest BLAST-derived bit score were chosen as the first two genes of a new cluster. If several pairs shared the same bit score, the pair with the shortest alignment length was selected first. New members were subsequently added to the cluster using agglomerative linkage clustering until no more suitable candidate genes were left. After completion of each cluster, all gene pairs having at least one clustered member gene were deleted from the gene pair list.

#### Estimation of K_S _values for gene pairs

In a first step the peptide sequences were aligned globally using *needle*. Afterwards, all positions containing a gap were removed from the alignment and the amino acids were replaced by their corresponding codons. The nucleotide alignment was used to calculate the K_S_-value with the maximum likelihood method implemented in *codeml *of the PAML package [70]. Codon frequencies were calculated from the average nucleotide frequencies at the three codon positions (codon frequency model F3 × 4). Because codeml can get stuck in suboptimal likelihood maxima, the calculation was repeated five times and the K_S_-value with the highest likelihood was then assigned to the gene pair.

#### Calculating the K_S _values used in the distribution

To remove node-connecting K_S _values > 5.0, subtree clustering based on the K_S_-values as distance measure was performed using average linkage clustering. Assuming that all genes in a resulting cluster with n members originate from the same ancestor gene, n-1 duplication events have taken place. However, the number of possible gene pairs or K_S_-values of a cluster with n members is n × (n-1)/2, which exceeds the number of duplication events for n > 2. Using all pairwise K_S_-values of a cluster directly in the age distribution would thus falsify it. Instead, we used approximate K_S_-values for the n-1 duplication events that were derived from the pairwise K_S_-values during the clustering. The merging steps taken during the K_S_-based clustering were represented in a bifurcating dendrogram. The terminal nodes represent the genes of the original cluster and each inner node represents the joining of two clusters, which also can be regarded as the duplication event giving rise to the two clusters. To each inner node, and each duplication event respectively, the average inter cluster K_S_-value of the merged gene clusters can be assigned. The inter cluster K_S_-values ≤ 5.0 were used to represent the duplication events of the cluster in the age distribution.

### Construction of linearized trees

We have inferred the age of *P. patens *duplicated genes by constructing linearized trees and comparing the time of gene duplication with the *A. thaliana*-poplar split or the monocot-eudicot split, following the method used by Vandepoele et al. [30]. To this end, the 22,237 protein sequences of *P. patens *were grouped into 1,967 gene families containing two to ten *P. patens *proteins based on sequence similarity [25]. All protein sequences of each gene family were used as queries to do BLASTP searches against proteins from *Oryza sativa *(TIGR release 4), *Populus trichocarpa *(JGI version 1, released June 7, 2006), *Arabidopsis thaliana *(TAIR release 6), *Chlamydomonas reinhardtii *(JGI release 3), and *Ostreococcus tauri *(released August 8, 2006). Gene families were built and neighbor-joining trees were constructed using LINTREE [29] based on the alignments of each gene family [30]. Only those gene families that included at least one outgroup sequence (*C. reinhardtii *or *O. tauri*) and sequences that could be used as reference or calibration points (see below) to estimate the date of the *P. patens *duplication, i.e. sequences from at least two different organisms out of the three angiosperm species (rice, poplar and *A. thaliana*), and which all had to have higher BLASTP scores than that of the outgroup sequence, were considered for further analyses. Linearized trees, which assume equal rates of evolution in different lineages of the tree [29], were constructed for each gene family after sequences evolving at highly deviated rates were removed [30]. The split of *A. thaliana *and poplar, or monocots (rice) and eudicots (*Arabidopsis *and poplar), set at 100 and 150 MYA, respectively, were used as reference points to estimate the age of the *P. patens *duplicates. Each node that was used for dating had to have bootstrap support ≥ 70% [71]. In cases where the tree had more than one reference point that could be used for dating, the duplication date was first calculated separately using each reference point. The tree was then discarded if the minimum and maximum date differed by >20 MYA. If the difference was ≤20 MY, the average of the date estimates from all possible reference points was taken as the date of the duplication event.

### Gene Ontology and pathway mapping

GO terms were assigned to the sequences using Blast2GO [44] with an E-value cutoff of 1E-25 and a minimal hit length of 80 amino acids. The GO Slim annotation (which avoids the redundancy of GO term association) was created using the generic GO Slim file, GO terms, definitions and ontologies [72]. It was determined (using five-fold leave-one-out cross validation) how many genes are necessary to do GO bias comparisons in order not to be affected by sampling bias. As it turned out, a sample size of at least 500 genes is sufficient to detect significantly biased categories. The fractions of genes assigned/devoid of individual GO terms were tested for deviation within the 765 duplicated genes in comparison with a randomly chosen reference set (excluding genes from the K_S _peak) of equal size (765 out of 2,202 possible genes) using Fisher's exact test. Resulting p values were adjusted to control for multiple testing by calculating the false discovery rate [73]. Statistics were performed with R 2.1.0 [74]. The KEGG pathways were assigned to the sequences representing the peaks using KAAS (KEGG Automatic Annotation Server 1.10; [75]). Searches were performed against the whole dataset with a bit score threshold of 60 and the bi-directional best hit method (BBH).

## Authors' contributions

SAR designed and supervised most of the research and wrote the paper, JI wrote the software and calculated the K_S _plot, JAF calculated the linearized trees, DL and AZ performed the GO and KEGG analyses, YVDP supervised part of the research and participated in writing the paper, RR participated in writing the paper.
