# Erratum to: An ancient genome duplication contributed to the abundance of metabolic genes in the moss *Physcomitrella patens*

**DOI:** 10.1186/s12862-016-0739-4

**Published:** 2016-09-08

**Authors:** Stefan A. Rensing, Julia Ick, Jeffrey A. Fawcett, Daniel Lang, Andreas Zimmer, Yves Van de Peer, Ralf Reski

**Affiliations:** 1 Plant Biotechnology, Faculty of Biology, University of Freiburg, Schaenzlestr. 1, D-79104 Freiburg, Germany; 2 Department of Plant Systems Biology, VIB, B-9052 Ghent, Belgium; 3 Bioinformatics and Evolutionary Genomics, Department of Molecular Genetics, Ghent University, Technologiepark 927, B-9052 Ghent, Belgium

## Erratum

The author’s would like to make the readers aware that the original version of this article [1] unfortunately contained a mistake. The following text published in the “Results and Discussion” section was supplied as follows:

"If we assume that the genome duplication indeed took place about 45 MYA (average of the peak in Fig. 1), and we assume that genes duplicated at that time have an average KS value of 0.85 (see Fig. 1), we can infer the rate of synonymous substitutions by simply dividing 0.85 by 45 MY. This gives us a rate of 1.9 synonymous substitutions per synonymous site per year, …"

However this should read:

"If we assume that the genome duplication indeed took place about 45 MYA (average of the peak in Fig. 1), and we assume that genes duplicated at that time have an average KS value of 0.85 (see Fig. 1), we can infer the rate of synonymous substitutions by simply dividing 0.85 by two times 45 MY (two times since Ks accumlate independently in the paralogs after the duplication). This gives us a rate of 9.4E-09 synonymous substitutions per synonymous site per year, …"
